# Pharmacokinetics of lopinavir/ritonavir in second-line treatment of children with HIV in the CHAPAS-4 trial

**DOI:** 10.1097/QAD.0000000000004328

**Published:** 2025-09-03

**Authors:** Anne E.M. Kamphuis, Timo Kiezebrink, Hylke Waalewijn, Alasdair Bamford, Alexander J. Szubert, Chishala Chabala, Mutsa Bwakura-Dangarembizi, Shafic Makumbi, Joan Nangiya, Vivian Mumbiro, Veronica Mulenga, Victor Musiime, Saskia N. de Wildt, Angela P.H. Colbers, Diana M. Gibb, David M. Burger

**Affiliations:** a Department of Pharmacy, Pharmacology & Toxicology, Radboud Research Institute for Medical Innovation (RIMI), Radboudumc; b Department of Pharmaceutical Sciences, Faculty of Science, University of Utrecht, Universiteitsweg 99, 3584 CG, Utrecht, the Netherlands; c Division of Clinical Pharmacology, Department of Medicine, University of Cape Town, Cape Town, South Africa; d Medical Research Council Clinical Trials Unit at University College London, Institute of Clinical Trials and Methodology; e Great Ormond Street Hospital for Children NHS Foundation Trust, London, UK; f Department of Paediatrics and Child Health, School of Medicine, University of Zambia; g University Teaching Hospital, Lusaka, Zambia; h University of Zimbabwe Clinical Research Centre, Harare, Zimbabwe; i Joint Clinical Research Centre, Mbarara; j Joint Clinical Research Centre; k Department of Paediatrics and Child Health, Makerere University, Kampala, Uganda; l Department of Intensive Care, Radboudumc; m Department of Neonatal and Pediatric Intensive Care, Erasmus MC Sophia Children's Hospital, Rotterdam, The Netherlands.

**Keywords:** children, HIV, lopinavir/ritonavir, pharmacokinetics, second-line

## Abstract

**Objective:**

Lopinavir/ritonavir (LPV/r) remains a much used drug combination for treatment of children with HIV, but pharmacokinetic data when the adult formulation (LPV/r 200/50 mg) is used for children weighing 25–34.9 kg, or when combined with tenofovir alafenamide/emtricitabine (TAF/FTC), is currently lacking.

**Design:**

We aim to provide this data by an intensive LPV/r pharmacokinetic sub-study nested within the CHAPAS-4 trial (#ISRCTN22964075).

**Methods:**

Children (3–15 years), weighing 14–24.9 kg received 200/50 mg LPV/r orally twice daily; those weighing 25–34.9 kg received 400/100 mg LPV/r in the morning and 200/50 mg in the evening; and those weighing at least 35 kg received 400/100 mg LPV/r twice daily. LPV/r was used in combination with either TAF/FTC or standard-of-care backbone (abacavir/lamivudine or zidovudine/lamivudine). Pharmacokinetic parameters were compared to those reported in children receiving WHO-recommended dosages.

**Results:**

We enrolled 40 children from Uganda, Zambia, and Zimbabwe. The geometric mean area under the concentration–time curve (AUC_0–12h_) for LPV was 116.2 h mg/l [coefficient of variation (CV%), 37%], comparable to children receiving WHO-recommended dosages. The geometric mean trough concentration was 7.7 mg/l (52%), 57% higher than the reference value of 4.9 mg/l (95% confidence interval, 4.14–5.80), mainly caused by higher exposure in children 25–34.9 kg. There were no differences in LPV AUC_0–12h_ or *C*_trough_ between backbones.

**Conclusion:**

Children (3–15 years), weighing at least 14 kg and taking LPV/r in second-line treatment achieve adequate exposure of LPV within limits reported to be safe and well tolerated. These data support the use of a LPV/r-based regimen and the adult formulation of 200/50 mg in children 25–34.9 kg.

## Introduction

In recent years, the number of children with HIV initiating treatment with antiretroviral therapy (ART) has risen. Improved access to HIV and viral load testing is increasing detection of first-line treatment failure as well [1,2]. Children with virological failure on an integrase strand transfer inhibitor (INSTI)-based regimen are recommended to switch to a protease inhibitor boosted by ritonavir [3]. Lopinavir/ritonavir (LPV/r) and atazanavir/ritonavir (ATV/r) are currently the recommended protease inhibitors as second-line treatment for children with HIV [3]. Although there are some disadvantages of LPV/r compared to ATV/r and darunavir/ritonavir (DRV/r), such as more gastrointestinal side-effects, higher risk of lipid abnormalities, poorer growth, and virological outcomes [4–6], LPV/r remains in many places the only available protease inhibitor in a pediatric formulation and was still being used by ~25% of children on ART in 2022 [7].

WHO dosing recommendations for LPV/r are based on weight bands. When treated with fixed dose combination tablets (available as 200/50 and 100/25 mg LPV/r), children 14–24.9 kg receive 200/50 mg twice daily, while those 25–34.9 kg receive 300/75 mg twice daily. The latter requires 100/25 mg tablets, as the film-coated tablets cannot be split or crushed, although limited availability in some countries raises questions about the utility and sustainability of this formulation [8]. A backbone of two nucleoside/nucleotide reverse transcriptase inhibitors (NRTIs) remains universally recommended for first-line and second-line ART. Abacavir (ABC) and zidovudine (ZDV) are still most commonly recommended in children in combination with lamivudine (3TC) [3]. Tenofovir alafenamide (TAF) combined with 3TC or emtricitabine (FTC) is listed as an alternative agent [3], although it was recently shown to be superior over ‘standard-of-care’ backbones in second line [6].

Pharmacokinetic data of LPV/r in children 25–34.9 kg treated with the adult 200/50 mg formulation, that is, 400/100 mg in the morning and 200/50 mg in the evening, as well as pharmacokinetic data when combined with TAF/FTC are lacking. Demonstrating adequate exposure with this dosing scheme facilitates use of adult LPV/r tablets in children 25–34.9 kg, simplifying procurement. We aim to close this information gap by an intensive LPV/r pharmacokinetic sub-study within the CHAPAS-4 trial (#ISRCTN22964075).

## Methods

CHAPAS-4 evaluated safety, efficacy, and pharmacokinetics of dolutegravir (DTG), DRV/r, LPV/r, and ATV/r in combination with an NRTI backbone (ZDV/3TC, ABC/3TC, or TAF/FTC) in children (3–15 years) from Zambia, Uganda, and Zimbabwe. The trial was approved by local and national ethical committees. Caregivers and participants (as applicable), provided informed consent and assent, respectively. Here, we report results of the LPV/r pharmacokinetic sub-study within CHAPAS-4. Results of the main trial and other pharmacokinetic sub-studies have been reported elsewhere [6,9–12].

Children weighing 14–24.9 kg received 200/50 mg LPV/r twice daily; those 25–34.9 kg received 400/100 mg in the morning and 200/50 mg in the evening; those at least 35 kg received 400/100 mg LPV/r twice daily. LPV/r tablet formulations and quantities administered are shown in the table in Supplemental Digital Content 1. Tablets could not be split or crushed. Dosing of NRTI backbones is shown in the table in Supplemental Digital Content 2.

After 6 weeks of treatment (at steady-state) the intensive 12-h pharmacokinetic assessment was performed. A standardized breakfast (~250 kcal, 5% fat) was provided 10 min before intake of the LPV/r morning dose. Co-medications that could influence the pharmacokinetics were not allowed within 2 h after ART intake. Blood samples were collected at *t* = 0 (predose) and 1, 2, 4, 6, 8, and 12 h postdose. Volumes of blood samples were within safety limits [13]. Details on laboratory procedures are described in Supplemental Digital Content 3.

Pharmacokinetic parameters were calculated using noncompartmental analysis (Phoenix 8.4 WinNonlin). Primary pharmacokinetic parameters were AUC_0–12_ _h_ (area under the concentration–time curve) and *C*_trough_ (12-h postdose concentration), which is related to LPV antiviral activity. We also reported the predose concentration (*C*_0_), as it may reflect the lowest level in children 25–34.9 kg. Other parameters determined were *C*_max_ (maximum plasma concentration), *T*_max_ (time to reach the maximum concentration), CL/F (apparent oral clearance), CL/F/kg (apparent oral clearance corrected for body weight), *V*_d_/*F* (apparent volume of distribution). and *T*_1/2_ (apparent elimination half-life).

The aim was to achieve pharmacokinetic parameters comparable to those in children who received WHO-recommended dosages in the KONCERT trial (see Table, Supplemental Digital Content 1), in which treatment with LPV/r BID demonstrated efficacy and low rates of viral rebound [14,15]. The target *C*_trough_ for LPV was 1.0  mg/l [16].

AUC_0–12_ _h_ and *C*_trough_ levels were compared between weight bands and NRTI backbones by one-way ANOVA on log-transformed values with Tukey post hoc analysis. Lipid elevation differences between weight bands were assessed by comparing changes in total, low-density lipoprotein (LDL), high-density lipoprotein (HDL) cholesterol, and triglycerides between week 0 and week 48 by one-way ANOVA. In addition, the correlation between LPV and RTV AUC_0–12_ _h_ and *C*_trough_ levels and changes in lipids levels was assessed.

## Results

Between February 2019 and September 2020, 51 children from Uganda, Zambia, and Zimbabwe were enrolled in this sub-study. Eleven children were excluded from analysis (for additional details, see Description Supplemental Digital Content 3). Of the children included in analysis, 11 received a backbone of ABC/3TC, 20 received TAF/FTC, and 9 received ZDV/3TC. Other demographic data are presented in Table 1.

**Table 1:** Patient demographics and summary of lopinavir pharmacokinetic parameters in children within CHAPAS-4 and reference values.

	Total CHAPAS-4 sub-study	Weight band				KONCERT
		14–19.9 kg 200/50 mg LPV/r (b.i.d.)	20–24.9 kg 200/50 mg LPV/r (b.i.d.)	25–34.9 kg 400/100 mg LPV/r AM, 200/50 mg LPV/r PM	35+ kg 400/100 mg LPV/r (b.i.d.)	
Number of participants (*N*)	40	9	10	9	12	53
Age (years)	10.3 (4.3–14.7)	5.9 (4.3–7.7)	9.7 (6.9–12.8)	10.2 (8.1–13.0)	13.7 (10.6–14.7)	11.0
Weight (kg)	25.4 (14.2–49.5)	18.0 (14.2–19.1)	22.8 (20.0–24.2)	27.0 (25.0–30.6)	43.6 (36.5–49.5)	31.0
Number of girls (% in WB)	17 (57%)	4 (44%)	7 (70%)	5 (56%)	7 (58%)	
Lopinavir						
AUC_0–12_ _h_ (h mg/l)	116.2 (37)	104.3 (38)	110.3 (33)	145.1 (40)	109.7 (32)	106.9 (97.8–116.9)
*C*_trough_ (mg/l)	7.7 (52)	6.2 (55)	6.9 (45)	11.9 (39)	7.0 (46)	4.9 (4.14–5.80)
*C*_0_ (mg/l)	8.1 (76)	9.1 (34)	8.0 (48)	6.5 (178)	8.9 (49)	n.a.
*C*_max_ (mg/l)	12.5 (32)	11.5 (30)	11.7 (32)	15.4 (44)	12.1 (28)	12.0 (11.1–12.9)
*T*_max_ (h)	4 (0–12)	4 (0–6)	4 (0–12)	6 (0–12)	4 (0–6.03)	n.a.
*T*_1/2_ (h)	10.5 (60)	10.5 (72)	9.0 (52)	15.1 (56)	8.3 (40)	n.a.
CL/*F* (l/h)	2.4 (46)	1.92 (38)	1.81 (33)	2.55 (38)	3.64 (32)	n.a.
CL/*F*/kg (l/h/kg)	0.09 (38)	0.11 (37)	0.08 (37)	0.09 (40)	0.09 (32)	0.089 (0.081–0.097)
*V*_d_/*F* (l)	38.4 (66)	27.4 (50)	23.9 (57)	72.7 (50)	49.3 (33)	n.a.
Ritonavir						
AUC_0–12_ _h_ (h mg/l)	5.8 (56)	4.6 (60)	4.8 (44)	7.8 (55)	6.2 (54)	5.9 (5.28–6.65)

Age and weight are presented as medians (range). Pharmacokinetic data are presented as geometric means (coefficient of variation for CHAPAS-4, 95% confidence interval for KONCERT), except for *T*_max_, which is presented as median (IQR). LPV/r denotes lopinavir-boosted ritonavir. AUC_0–12_ _h_, the area under the concentration–time curve from 0 to 12 h; b.i.d., twice daily; *C*_0_, predose concentration; CL/F, apparent oral clearance; CL/F/kg, apparent oral clearance corrected for body weight; *C*_max_, maximum plasma concentration; *C*_trough_, concentration 12 h after dosing; *T*_1/2_, elimination half-life; *T*_max_, time to reach the maximum plasma concentration; *V*_d_/*F*, apparent volume of distribution.

**Fig. 1:**
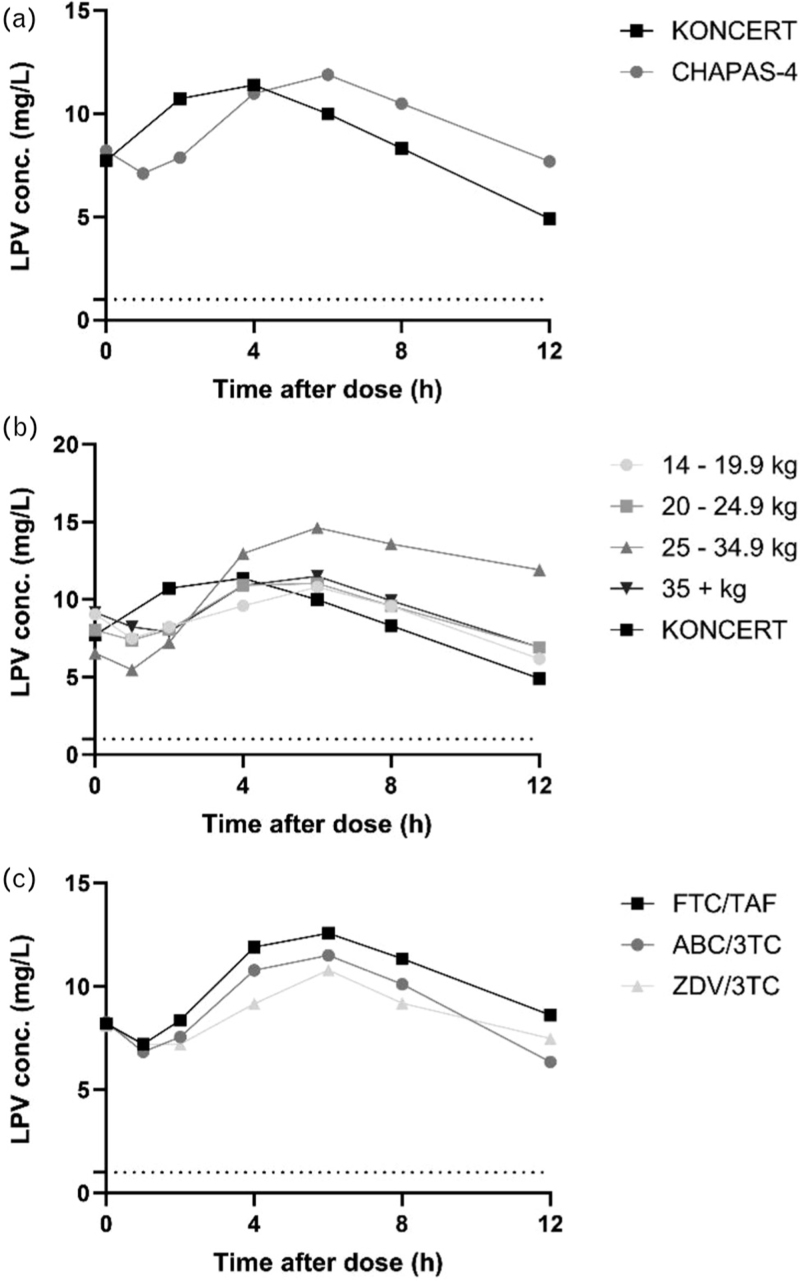
Geometric mean lopinavir plasma concentrations versus time profiles of the CHAPAS-4 pharmacokinetic sub-study compared to the KONCERT trial (a); the CHAPAS-4 sub-study by weight band (b); and the CHAPAS-4 sub-study by NRTI backbone (c).

LPV pharmacokinetic parameters for all children are summarized in Table 1. The geometric mean AUC_0–12h_ and geometric mean *C*_max_ are comparable to that of children in KONCERT [14]. The geometric mean *C*_trough_ is ~57% higher than the geometric mean value of children in KONCERT. The geometric mean LPV plasma concentration versus time profile for all children in this sub-study and for KONCERT are shown in Fig. 1a.

The horizontal dotted line indicates the LPV target *C*_trough_ of 1 mg/l. LPV denotes lopinavir. ABC/3TC, abacavir/lamivudine; FTC/TAF, emtricitabine/tenofovir alafenamide; ZDV/3TC, zidovudine/lamivudine.

LPV pharmacokinetic parameters for each weight band are summarized in Table 1. AUC_0–12_ _h_ values did not significantly differ by weight band (one-way ANOVA, *P* = 0.189). Comparison of *C*_trough_ values did show a difference between weight bands (*P* = 0.015). The *C*_trough_ was higher in children weighing 25–34.9 kg compared to children in the lowest and highest weight band (Tukey post hoc analysis, *P* = 0.021 and 0.048, respectively). Despite differences, all 40 individual *C*_trough_ values were above the target of 1.0 mg/l. *C*_0_ values of LPV did not differ by weight band (*P* = 0.674). One participant weighing 25–34.9 kg had a *C*_0_ value below the target of 1.0 mg/l. Geometric mean LPV plasma concentrations versus time profiles by weight band and for KONCERT are shown in Fig.1b. Changes in total, HDL, LDL cholesterol, and triglycerides between week 0 and week 48 did not differ by weight band (*P* = 0.230, 0.395, 0.462, and 0.612, respectively) (see Figure, Supplemental Digital Content 4). In addition, no correlation was found between AUC_0–12_ _h_ or *C*_trough_ values of LPV or RTV and lipid changes between weeks 0 and 48.

There were no differences in LPV AUC_0–12_ _h_ and *C*_trough_ between backbones (one-way ANOVA, *P* = 0.331 and 0.293, respectively). Geometric mean LPV plasma concentrations versus time profiles by NRTI backbone are shown in Fig. 1c.

RTV AUC_0–12_ _h_ values for all children and each weight band are summarized in Table 1. The GM AUC_0–12_ _h_ was comparable to what was observed in KONCERT. AUC_0–12_ _h_ values of RTV did not differ by weight band (one-way ANOVA, *P* = 0.106).

## Discussion

This study demonstrated that a dose of 200/50 mg LPV/r twice daily in children 14–24.9 kg, 400/100 mg LPV/r in the morning, and 200/50 mg LPV/r in the evening in children 25–34.9 kg and 400/100 mg LPV/r twice daily in children at least 35 kg, in combination with a backbone of TAF/FTC or SOC, achieve AUC_0–12_ _h_ levels of LPV comparable to reference values found to be well tolerated and effective in children [14–17]. The geometric mean *C*_trough_ observed in this sub-study is ~57% higher compared to the reference value.

These results are reflected by the LPV plasma concentration time curves (Fig. 1a). The higher overall *C*_trough_ is mainly caused by higher concentrations measured in children 25–34.9 kg, although *C*_trough_ values in the other weight bands are also slightly higher than observed in KONCERT (Fig. 1b). In addition, the *T*_max_ of LPV is later compared to the reference value. Children in this sub-study received LPV/r after breakfast, while children in KONCERT received LPV/r before breakfast or under fasting conditions. This might cause LPV absorption to be delayed in children in this sub-study, resulting in a later *T*_max_ and higher *C*_trough_ values compared to the KONCERT trial.

Although not statistically significant, the AUC_0–12_ _h_ seems slightly higher in children 25–34.9 kg compared to the other weight bands. This could be explained by the relatively higher milligram per kilogram body weight dosing in this weight band in the morning, that is, 400/100 mg LPV/r. Results from the main trial showed that treatment with LPV/r was associated with less favorable lipid profiles compared to treatment using DTG, DRV/r, or ATV/r [6]. The higher LPV exposure during the day in children 25–34.9 kg might raise concerns for increased risk of toxicity compared with the other weight bands. However, comparison of lipid changes between weeks 0 and 48 within this sub-study revealed no differences between weight bands. Additionally, no correlation was found between AUC_0–12_ _h_ or *C*_trough_ values of both LPV and RTV and the extent of lipid changes, indicating no increased risk of lipid abnormalities in children weighing 25–34.9 kg compared with the other weight bands. Studies in adults show conflicting results, as some did not find an association between LPV levels and lipid elevations [18,19], while others showed a correlation between LPV *C*_trough_ levels and increased lipid levels [20,21]. Of note, in CHAPAS-4, lipids were not measured during the same visit as the pharmacokinetic assessments. Additionally, a pediatric study on pharmacokinetics of high doses LPV/r reported a median *C*_12_ _h_ of 12.4 mg/l (range, 2.88–12.6) and a median AUC_0–12_ _h_ of 162.2 h mg/l (range, 63.8–185.7) in children on LPV/r with at least two NRTIs, which is comparable to values in children 25–34.9 kg observed in this sub-study [17]. The high doses were well tolerated, with no withdrawals for gastrointestinal side-effects, no gastrointestinal toxicity greater than grade 2, and no relationship between drug concentrations and heart rate or QTc. A more detailed pharmacokinetic/pharmacodynamic (PK/PD) analysis will be conducted to evaluate the relationship between lopinavir exposure and the occurrence of adverse events.

Given LPV levels in this sub-study are within limits reported to be safe and well tolerated, these data support use of the 200/50 mg LPV/r formulation in children 25–34.9 kg. Considering low availability of 100/25 mg LPV/r in some countries [8], this is a potential advantage that could overcome issues regarding stock-outs of pediatric formulations and consequently treatment disruption.

AUC_0–12_ _h_ and *C*_trough_ values of LPV did not differ between NRTI backbones, indicating that TAF/FTC does not affect the pharmacokinetics of LPV after administration of LPV/r. This is consistent with a study in adults investigating potential drug interactions between TAF and several antiretroviral drugs [22].

RTV AUC_0–12_ _h_ observed in children in this sub-study is comparable to the AUC_0–12_ _h_ observed in KONCERT. Although not statistically significant, RTV AUC_0–12_ _h_ seems slightly higher in children 25–34.9 kg compared to the other weight bands. This is in line with observations for LPV and is explained by the higher milligram per kilogram body weight morning dose of RTV in this weight band.

In conclusion, results of this sub-study show that children 3–15 years, taking LPV/r in second-line treatment, achieve adequate concentrations of LPV. This is in line with main efficacy and safety results of CHAPAS-4 [6]. If DTG, ATV/r, and DRV/r are not available or indicated, these data support the use of a LPV/r-based regimen as second-line treatment for children from at least 14 kg, including the adult formulation of 200/50 mg LPV/r in children 25–34.9 kg and in combination with TAF/FTC.

## Acknowledgements

D.M.G. conceived the study, the CHAPAS-4 trial team conducted the clinical trial, A.S. managed the trial data. A.C., D.M.B., and H.W. led the pharmacokinetic sub-study. A.E.M., T.K., and A.C. conducted the noncompartmental analysis. C.C., M.B., S.M. J.N. V.Mul., and V. Mus. carried out the trial activities. H.W., A.B., S.N.W., A.C., and D.M.B. critically reviewed and provided input on the manuscript. All authors read and approved the final manuscript.

We thank the participants of the CHAPAS-4 trial and their families, the principal investigators, and their staff at all the centers participating in the CHAPAS-4 trial, and the technicians of the Department of Pharmacy of Radboudumc.

The CHAPAS-4 trial is sponsored by University College London (UCL), with central management by the Medical Research Council (MRC) Clinical Trials Unit at UCL, supported by MRC core funding (MC_UU_00004/03). The main funding for this study is provided by the European and Developing Countries Clinical Trials Partnership (EDCTP; TRIA2015-1078). Additional funding is received from ViiV Healthcare, Janssen Pharmaceuticals, and Gilead Sciences Inc.

### Conflicts of interest

A.B. is chair of the Penta/EACS Paediatric HIV Treatment Guidelines Working Group and has received fixed-term consultancy fees from the WHO-hosted Global Accelerator for Paediatric Formulations (GAP-f). A.C. has received honoraria from Merck Sharp & Dohme and Gilead (fees paid to institution) and has received study grants from MSD, Gilead Sciences and ViiV Healthcare. D.B. has received research grants from ViiV Healthcare, Merck, and Gilead Sciences; payments from ViiV Healthcare and Gilead Sciences for serving on advisory boards; payment from ViiV Healthcare for speaking at symposia; payment or honoraria for lectures from Pfizer and Gilead Sciences and for advisory board for Merck; and is the co-founder of Global DDI Solutions. H.W. received consulting fees for an unrelated WHO project and consulting fees from European AIDS Clinical Society (EACS) as member of the guideline committee paediatric section of EACS HIV treatment guideline. S.N.W.'s employer receives compensation for DSMB membership from Khomdrion. All other authors declare no potential conflicts of interest.

## Supplementary Material

Supplemental Digital Content

## Supplementary Material

Supplemental Digital Content

## Supplementary Material

Supplemental Digital Content

## Supplementary Material

Supplemental Digital Content
