# Supplementary material for: Pharmacokinetics of lopinavir/ritonavir in second-line treatment of children with HIV in the CHAPAS-4 trial
Source: AIDS. 2025 Sep 3;39(15):2254–9. doi: 10.1097/QAD.0000000000004328 (PMC12629111; doi:10.1097/QAD.0000000000004328)
Supplement: Supplemental Digital Content [file aids-39-2254-s001.docx]

**Title:**

Pharmacokinetics of lopinavir/ritonavir in second-line treatment of children with HIV in the CHAPAS-4 trial

**Corresponding author:**

Anne Elisa Maria Kamphuis, MSc, PharmD

Department of Pharmacy, Pharmacology & Toxicology, Radboud Research Institute for Medical Innovation (RIMI), Radboudumc, The Netherlands

Geert Grooteplein Zuid 10, 6525 GA Nijmegen, The Netherlands,

E-mail: [Anne.Kamphuis@radboudumc.nl](mailto:Anne.Kamphuis@radboudumc.nl)

Tel: +31 (0) 631018525

**Supplemental Digital content 1**

***Table 1.*** *Formulations and number of LPV/r tablets administered to CHAPAS-4 participants for the different weight bands.*

| **LPV/r** | | | | | |
| --- | --- | --- | --- | --- | --- |
| **CHAPAS-4** | | **Daily dose** | **KONCERT** | | **Daily dose** |
| **Weight band** | **200/50 mg**  **BD (AM+PM)** |  | **Weight band** | **100/25 mg**  **BD (AM+PM)** |  |
| *14 – 19.9 kg** | 1+1 | 400/100 mg | *≥15 - ≤25 kg* | 2+2 | 400/100 mg |
| *20 – 24.9 kg* | 1+1 | 400/100 mg |  |  |  |
| *25 – 34.9 kg* | 2+1 | 600/150 mg | *>25 - ≤35 kg* | 3+3 | 600/150 mg |
| *35+ kg* | 2+2 | 800/200 mg | *>35 kg* | 4+4 | 800/200 mg |

**If 100/25 mg tablets were used for children in this weight band, a double dose of the tablets was given (i.e. 2+2)*
