# Supplementary material for: Pharmacokinetics of lopinavir/ritonavir in second-line treatment of children with HIV in the CHAPAS-4 trial
Source: AIDS. 2025 Sep 3;39(15):2254–9. doi: 10.1097/QAD.0000000000004328 (PMC12629111; doi:10.1097/QAD.0000000000004328)
Supplement: Supplemental Digital Content [file aids-39-2254-s002.docx]

**Title:**

Pharmacokinetics of lopinavir/ritonavir in second-line treatment of children with HIV in the CHAPAS-4 trial

**Corresponding author:**

Anne Elisa Maria Kamphuis, MSc, PharmD

Department of Pharmacy, Pharmacology & Toxicology, Radboud Research Institute for Medical Innovation (RIMI), Radboudumc, The Netherlands

Geert Grooteplein Zuid 10, 6525 GA Nijmegen, The Netherlands,

E-mail: [Anne.Kamphuis@radboudumc.nl](mailto:Anne.Kamphuis@radboudumc.nl)

Tel: +31 (0) 631018525

**Supplemental Digital content 2**

***Table 2****. Daily dose of NRTI backbones administered to CHAPAS-4 participants for the different weight bands.*

| **Weight band** | **FTC/TAF** | | **ABC/3TC** | | **ZDV/3TC** | |
| --- | --- | --- | --- | --- | --- | --- |
|  | *120/15 mg*  *QD* | *200/25 mg*  *QD* | *120/60 mg*  *QD* | *600/300 mg*  *QD* | *60/30 mg*  *BD (AM+PM)* | *300/150 mg*  *BD (AM+PM)* |
| *14 – 19.9 kg* | 1 |  | 2.5 |  | 3+2** |  |
| *20 – 24.9 kg* | 1 |  | 3 |  | 3+3 |  |
| *25 – 34.9 kg* |  | 1 |  | 1* |  | 1+1 |
| *35+ kg* |  | 1 |  | 1* |  | 1+1 |

*** Or 2.5 + 2.5 if tablets were scored*

** Or 5 tablets of ABC/3TC 120/60 mg*
