# Supplementary material for: Pharmacokinetics of lopinavir/ritonavir in second-line treatment of children with HIV in the CHAPAS-4 trial
Source: AIDS. 2025 Sep 3;39(15):2254–9. doi: 10.1097/QAD.0000000000004328 (PMC12629111; doi:10.1097/QAD.0000000000004328)
Supplement: Supplemental Digital Content [file aids-39-2254-s003.docx]

**Title:**

Pharmacokinetics of lopinavir/ritonavir in second-line treatment of children with HIV in the CHAPAS-4 trial

**Corresponding author:**

Anne Elisa Maria Kamphuis, MSc, PharmD

Department of Pharmacy, Pharmacology & Toxicology, Radboud Research Institute for Medical Innovation (RIMI), Radboudumc, The Netherlands

Geert Grooteplein Zuid 10, 6525 GA Nijmegen, The Netherlands,

E-mail: [Anne.Kamphuis@radboudumc.nl](mailto:Anne.Kamphuis@radboudumc.nl)

Tel: +31 (0) 631018525

**Supplemental Digital content 3**

*Procedures on PK sample processing*

Blood samples were processed within 1 hour of collection. Plasma was stored at -80 °C, before shipment to the central laboratory for quantitative analysis. LPV and ritonavir (RTV) concentrations were measured using a validated high performance liquid chromatography bioanalytical quantification method with a lower limit of quantification of 0.105 mg/L for LPV and 0.045 mg/L for RTV. The assay was validated through the International Interlaboratory Quality Control Program for Measurement of Antiretroviral Drugs and plasma, and the Clinical Pharmacology Quality assurance program. [1, 2]

*Exclusion of participants from analysis*

Eleven children were excluded from pharmacokinetic analysis due to non-adherence (n=5), samples that did not arrive (n=3), missing data due to swapped samples in the laboratory (n=1), use of concomitant medication that was not allowed (n=1), and administration of the wrong dose (n=1).

**References**

1. *University at Buffalo, Clinical Pharmacology Quality Assurance Program.*; Available from: <https://www.buffalo.edu/tprc/section-2/CPQA.html>.

2. *Association for Quality Assessment and Clinical Toxicology (KKGT), Dutch Foundation for Quality Assessment in Medical Laboratories (SKML)*. Available from: <https://www.skml.nl/en/schemes/125/>.
