# Supplementary material for: Pharmacokinetics of lopinavir/ritonavir in second-line treatment of children with HIV in the CHAPAS-4 trial
Source: AIDS. 2025 Sep 3;39(15):2254–9. doi: 10.1097/QAD.0000000000004328 (PMC12629111; doi:10.1097/QAD.0000000000004328)
Supplement: Supplemental Digital Content [file aids-39-2254-s004.docx]

**Title:**

Pharmacokinetics of lopinavir/ritonavir in second-line treatment of children with HIV in the CHAPAS-4 trial

**Corresponding author:**

Anne Elisa Maria Kamphuis, MSc, PharmD

Department of Pharmacy, Pharmacology & Toxicology, Radboud Research Institute for Medical Innovation (RIMI), Radboudumc, The Netherlands

Geert Grooteplein Zuid 10, 6525 GA Nijmegen, The Netherlands

E-mail: [Anne.Kamphuis@radboudumc.nl](mailto:Anne.Kamphuis@radboudumc.nl)

Tel: +31 (0) 631018525

**Supplemental Digital content 4**


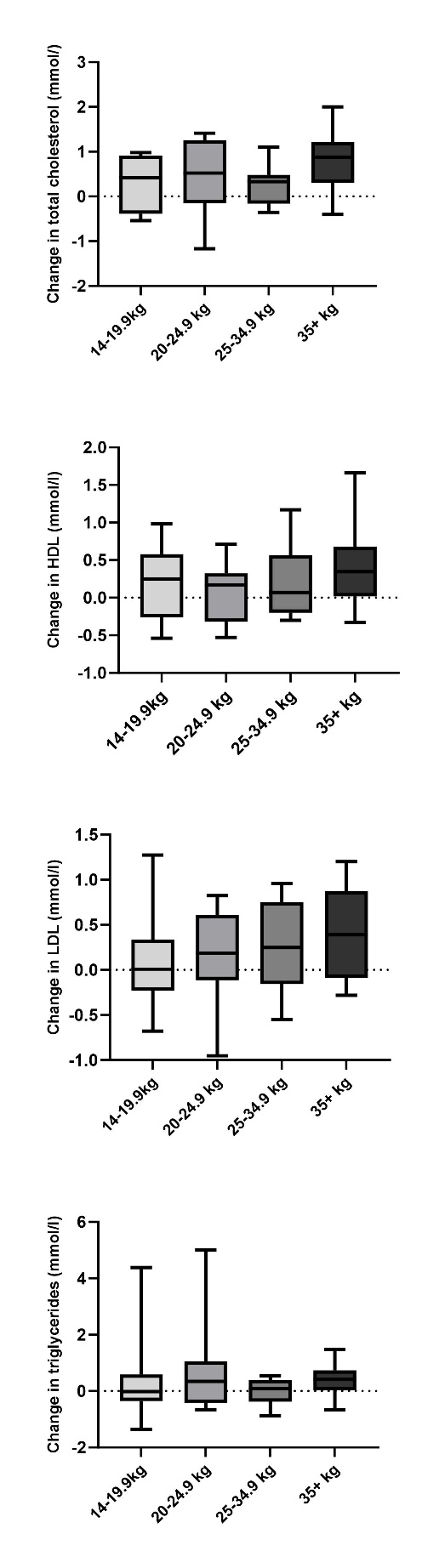


*Figure 1. Boxplots depicting changes of total (I), HDL (II), LDL (III) cholesterol and triglycerides (IIII) between week 0 and week 48 stratified by weight band.*
